# Quantitative DNA methylation analyses reveal stage dependent DNA methylation and association to clinico-pathological factors in breast tumors

**DOI:** 10.1186/1471-2407-13-456

**Published:** 2013-10-05

**Authors:** Jovana Klajic, Thomas Fleischer, Emelyne Dejeux, Hege Edvardsen, Fredrik Warnberg, Ida Bukholm, Per Eystein Lønning, Hiroko Solvang, Anne-Lise Børresen-Dale, Jörg Tost, Vessela N Kristensen

**Affiliations:** 1 Department of Clinical Molecular Biology and Laboratory Science (EpiGen), Akershus University hospital, Division of Medicine, 1476 Lørenskog, Norway; 2 Institute for Clinical Medicine, Faculty of Medicine, University of Oslo, 0372 Oslo, Norway; 3 Department of Genetics, Institute for Cancer Research, OUS Radiumhospitalet Montebello, 0310 Oslo, Norway; 4 Laboratory for Epigenetics, Centre National de Génotypage, CEA – Institut de Génomique, 91000 Evry, France; 5 Department of Surgery, Uppsala University Hospital, Uppsala, Sweden; 6 Department of Surgery, Akerhus University Hospital, Oslo, Norway; 7 Section of Oncology, Institute of Medicine, University of Bergen and Department of Oncology, Haukeland University Hospital, N5021 Bergen, Norway

**Keywords:** Breast cancer, DNA methylation, Methylation index, Stage, TP53

## Abstract

**Background:**

Aberrant DNA methylation of regulatory genes has frequently been found in human breast cancers and correlated to clinical outcome. In the present study we investigate stage specific changes in the DNA methylation patterns in order to identify valuable markers to understand how these changes affect breast cancer progression.

**Methods:**

Quantitative DNA methylation analyses of 12 candidate genes *ABCB1*, *BRCCA1*, *CDKN2A*, *ESR1*, *GSTP1*, *IGF2*, *MGMT, HMLH1*, *PPP2R2B*, *PTEN, RASSF1A* and *FOXC*1 was performed by pyrosequencing a series of 238 breast cancer tissue samples from DCIS to invasive tumors stage I to IV.

**Results:**

Significant differences in methylation levels between the DCIS and invasive stage II tumors were observed for six genes *RASSF1A, CDKN2A, MGMT, ABCB1, GSTP1* and *FOXC1*. *RASSF1A*, *ABCB1* and *GSTP1* showed significantly higher methylation levels in late stage compared to the early stage breast carcinoma. Z-score analysis revealed significantly lower methylation levels in DCIS and stage I tumors compared with stage II, III and IV tumors. Methylation levels of *PTEN, PPP2R2B, FOXC1, ABCB1* and *BRCA1* were lower in tumors harboring *TP53* mutations then in tumors with wild type *TP53*. Z-score analysis showed that *TP53* mutated tumors had significantly lower overall methylation levels compared to tumors with wild type *TP53*. Methylation levels of *RASSF1A*, *PPP2R2B*, *GSTP1* and *FOXC1* were higher in ER positive vs. ER negative tumors and methylation levels of *PTEN* and *CDKN2A* were higher in HER2 positive vs. HER2 negative tumors. Z-score analysis also showed that HER2 positive tumors had significantly higher z-scores of methylation compared to the HER2 negative tumors. Univariate survival analysis identifies methylation status of *PPP2R2B* as significant predictor of overall survival and breast cancer specific survival.

**Conclusions:**

In the present study we report that the level of aberrant DNA methylation is higher in late stage compared with early stage of invasive breast cancers and DCIS for genes mentioned above.

## Background

Breast cancer is the most common form of malignant disease in women worldwide and also the principal cause of death from cancer among women globally. In Norwegian women breast cancer accounts for approximately 23% of all cancers and ~800 women die as a result of the disease every year. Breast cancer is a heterogeneous disease with distinct histopathological, genetic and epigenetic characteristics. Epigenetic regulation is critical for normal growth and development and provides a layer of transcriptional control. Epigenetic alterations which occur in transformed cells involve changes in DNA methylation including global hypomethylation and focal hypermethylation, histone modifications and nucleosomal remodeling [1]. Epigenetic changes are considered to be an early event in tumor development and one of the hallmarks of cancer [2]. The degree of DNA methylation in the promoter region of tumor suppressor genes, transcription factors and drug response genes may play a role in the initiation of cancer, tumor progression and response to treatment. Identification of early epigenetic changes in breast cancer might give valuable markers for early detection and contribute to the understanding of how these changes affect the progression of the disease and prognosis for the patient. We previously described treatment-specific DNA methylation patterns in 432 CpGs in the promoter regions of 14 genes in samples from 75 patients with locally advanced breast cancer treated with doxorubicin [3]. Further, we identified four novel genes (*ABCB1, FOXC1, PPP2R2B* and *PTEN)* that were found to be already aberrantly methylated in DCIS (Ductal carcinoma *in situ*), a pre-invasive stage of breast cancer [4] and that were also found to be methylated in the locally advanced breast cancers [3]. These findings raised the question if DNA methylation patterns evolve between the different stages of breast cancers and pre-neoplastic lesions or are similar and independent of tumor stage. In the present study, using the same candidate gene approach as previously described [3,4], we performed a large-scale analysis of 12 candidate genes and determined quantitatively the DNA methylation patterns of the 12 genes in 238 breast cancer patients of all stages from early premalignant DCIS to advanced metastatic disease. In addition to the analysis of stage dependent DNA methylation patterns, associations between additional clinico-pathological factors of breast cancer such as grade and ER status and DNA methylation patterns of these 12 genes were assessed.

## Methods

### Patient material

A total of 238 samples were included in present study: 75 patients with locally advanced breast cancer that were enrolled in a prospective study evaluating predictive factors for response to doxorubicin [3] and 35 patients treated with 5-FU and Mitomycin for locally advanced breast cancer [5], patients in both cohorts were admitted to the Haukeland University Hospital in Norway between 1991 and 2001; 57 samples from a series of 212 breast cancers samples collected from Ullevål University Hospital (Norway) between 1990 and 1994 [6]; 71 tumors from a population-based cohort including 854 women diagnosed between 1986 and 2004 in Uppsala, Sweden with either: a) pure ductal carcinoma *in situ* of the breast (DCIS), b) an invasive breast cancer, 15 mm or less, without an *in situ* component or c) a mixed lesion, i.e., a lesion with both an invasive- and an *in situ* component [4]. Clinical and molecular characteristics of the tumors are given in Table 1. DNA from six normal breast tissues was included to identify the DNA methylation baseline in normal tissues. Normal breast tissue was obtained from women who underwent a biopsy of the mammary gland because of mammographic screening and for whom histology confirmed the presence of only normal tissue. All patients had given informed consent, and the project was approved by the local ethical committee.

**Table 1:** Clinical characteristics of the analyzed samples

**Clinicopathological factor**	**N(%)**
**Tumorsize**	
**T0**	**26(11%)**
**T1**	**67(28.3%)**
**T2**	**35(14.8%)**
**T3**	**64(27%)**
**T4**	**45(18.9%)**
**Unknown**	**1**
**Stage**	
**DCIS**	**26(11.5%)**
**I**	**65(28.9%)**
**II**	**20(8.9%)**
**III**	**92(40.9%)**
**IV**	**22(9.8%)**
**Unknown**	**13**
**Lymphnode status**	
**N0**	**114(50.7%)**
**N1**	**76(34.9%)**
**N2**	**32(14.4%)**
**Unknown**	**16**
**Grade**	
**G1**	**42(17.7%)**
**G2**	**128(54%)**
**G3**	**67(28.3%)**
**Unknown**	**1**
**HER2 status**	
**Negative**	**60(57.1%)**
**Positive**	**45(42.9%)**
**Unknown**	**133**
**Progesteron receptor**	
**Negative**	**69(29.2%)**
**Positive**	**167(70.8%)**
**Unknown**	**2**
**Estrogene receptor**	
**Negative**	**53(22.7%)**
**Positive**	**180(77.3%)**
**Unknown**	**5**
**TP53 mutation**	
**Wild type**	**174(73.4%)**
**Mutated**	**63(26.6%)**
**Unknown**	**1**
**Distant metastasis**	
**No distant metastasis**	**214(90.3%)**
**Distant metastasis**	**23(9.7%)**
**Unknown**	**1**
**Molecular subtypes**	
**Luminal A**	**52(30.8%)**
**Luminal B**	**34(20.1%)**
**ERRB2**	**29(17.2%)**
**Basal**	**37(21.9%)**
**Normal**	**17(10%)**
**Unknown**	**69**

Unknown data was excluded from total when the percentage was calculated.

### Methylation assays

DNA concentrations were determined using the Quant-iT™ dsDNA broad range assay kit (Invitrogen, Cergy Pontoise, France) and normalized to a concentration of 50 ng/μl. One μg of DNA was bisulphite converted using the MethylEasy™ HT Kit for Centrifuge (Human Genetic Signatures, North Ryde, Australia) according to the manufacturer’s instructions. Quantitative DNA methylation analysis of the bisulphite treated DNA was performed by pyrosequencing or - in case of several sequencing primers - by serial pyrosequencing [7]. Oligonucleotides for PCR amplification and pyrosequencing (Additional file 1) were synthesized by Biotez (Buch, Germany) [3]. In the present study, same candidate gene approach was used as previously described [3,4] with the difference in number of covered CpGs (205 in our case) because of absence of variability. These genes were initially selected on the following basis: previous reports of DNA methylation in breast tumors or at least breast cancer cell lines (*ABCB1*[8], *BRCA1*[9], *CDKN2A*[9], *ESR1*[10], *GSTP1*[11], *IGF2*[12], *MGMT*[9], *MLH1*[9], *PPP2R2B*[13], *PTEN*[14], *RASSF1A*[15]) and genes displaying variation in breast cancer gene expression profiles (*FOXC1*[16]).

### Statistical analysis

The average value of methylation for all CpGs in a target region was calculated for each sample and each gene. Although there was some stochastic variation between different CpG positions, the overall methylation level was quite constant and CpG positions were highly correlated in the analyzed regions. A sample was considered 1) hypermethylated if the percentage of DNA methylation was higher than the sum of two times the standard deviation and mean of the normal samples, 2) normal-like methylation if the% DNA methylation was in range of two times the standard deviation +/− mean of the normal sample and 3) hypomethylated if% DNA methylation was lower than two times standard deviation – mean of normal sample. The aggregated quantitative DNA methylation data is presented in Table 2. Differences in the presence of methylation were determined by a two-sided Fisher test (for variables with two categories) and Chi- squared tests for variables with three or more categories. Odds ratio and 95% confidence intervals were calculated for two-categorical variables. Differences in the distribution of methylation were assessed by the non-parametric Mann–Whitney test (on parameters with 2 categories) or the Kruskal-Wallis test analysis on parameters with more than two categories. All obtained p-values were corrected with the Bonferroni correction method in which the p-values are multiplied by the number of comparisons. The methylation index of samples (Z-score) was calculated as: (methylation level of each sample – mean value of methylation levels)/SD of methylation levels. Then the sum for the 12 genes was calculated giving one single value (Z-score) for each sample. The false discovery rate was not considered in this study due to small number of genes which were tested. All calculations were performed using Statistical Package for Science version 18.

**Table 2:** Quantitative methylation data

**Gene**	**Mean of samples**	**SD of samples**	**Mean of normal**	**SD of normal**
*BRCA1*	85.36	11.32	85.48	6.15
*RASSF1A*	30.92	17.34	3.37	0.99
*PTEN*	5.15	4.07	2.83	1.28
*PPP2R2B*	10.83	9.80	3.18	1.17
*CDKN2A*	4.68	4.64	3.58	1.08
*MLH1*	2.94	3.85	2.72	0.47
*MGMT*	4.21	3.53	4.48	1.11
*ABCB1*	16.18	17.25	2.63	1.23
*IGF2*	38.28	9.13	40.7	2.41
*GSTP1*	17.18	17.27	3.73	1.01
*FOXC1*	13.41	13.23	3.6	1.98
*ESR1*	2.92	2.50	4.43	2.94

Univariate, Kaplan-Meier analyses and the log-rank test for each parameter for single gene was performed to investigate which genes and parameters affect survival. Further, multivariate, the Cox proportional hazard model was used to identify independent prognostic markers for all genes and from all clinical parameters: age, stage, tumor size and grade, lymph node status, *TP53* mutation status, ER, PR status, T status. Methylation status of all genes was treated as continuous and categorical. We constructed possible model candidates using all combinations of given variables. To select the best-fitted model from all candidates, we evaluated the Akaike Information Criterion (AIC) [17]**.** AIC gives an evaluation for model selection, which is modified by a penalty increasing with the number of variables of the model. Analyzing all combinations of given variables we selected the model that fitted best to the data indicated by a minimal value for the AIC.

## Results

### Methylation analysis and correlation with clinico-pathological parameters: stage and grade

A total of 48790 epigenotypes were generated through analyses of 205 CpGs in 12 genes (*ABCB1* (20 CpGs), *BRCA1* (19 CpGs), *CDKN2A* (28 CpGs), *ESR1* (21 CpGs), *FOXC1* (9 CpGs), *GSTP1* (21 CpGs), *IGF2* (17 CpGs), *MGMT* (9 CpGs), *MLH1* (16 CpGs), *PPP2R2B* (14 CpGs), *PTEN* (19 CpGs) and *RASSF1A* (12 CpGs)). Six normal samples were used to estimate the normal-like methylation levels for all analyzed genes. Our analysis showed that five genes *ABCB1, FOXC1, GSTP1*, *PPP2R2B* and *RASSF1A* were the most frequently hypermethylated genes in all invasive samples as well as in the DCIS samples. *PTEN* was hypermethylated in invasive cancer of stage II, III and IV and *MGMT* was hypomethylated in invasive tumors of stage II, III and IV. *CDKN2A* had a normal-like methylation level in a high percentage of the DCIS samples and early stage tumors. In late stage tumors *CDKN2A* showed higher percentage of hypermethylated samples compared to the early stage tumors and the DCIS. The methylation levels of *ESR1* and *MLH1* were normal-like both in the DCIS and the invasive tumors and *BRCA1* had a normal-like methylation level in almost all the DCIS and all invasive tumors (Table 3.).

**Table 3:** Methylation status of 12 genes in normal tissue, DCIS and invasive breast cancer patients

		**Normal**		**DCIS**		**Invasive**							
						**I**		**II**		**III**		**IV**	
**GENE**	**Methylation status**	**N**	**%**	**Sample number**	**%**	**Sample number**	**%**	**Sample number**	**%**	**Sample number**	**%**	**Sample number**	**%**
***ABCB1***	**Hypomethylated**	**0**	**0**	**0**		**0**		**0**		**0**		**0**	
	**Normal like**	**6**	**100**	**15**	**57.7**	**33**	**52.4**	**6**	**30**	**31**	**36**	**6**	**30**
	**Hypermethylated**	**0**		**11**	**42.3**	**30**	**47.6**	**14**	**70**	**57**	**64**	**14**	**70**
***BRCA1***	**Hypomethylated**	**0**	**0**	**0**	**0**	**2**	**3**	**2**	**10**	**10**	**12.8**	**1**	**5.9**
	**Normal like**	**6**	**100**	**26**	**100**	**63**	**97**	**18**	**90**	**68**	**87.2**	**16**	**94.1**
	**Hypermethylated**	**0**		**0**	**0**	**0**		**0**		**0**		**0**	
***CDKN2A***	**Hypomethylated**	**0**	**0**	**0**	**0**	**0**		**0**		**0**		**0**	
	**Normal like**	**6**	**100**	**26**	**100**	**60**	**92.3**	**18**	**90**	**61**	**67.8**	**14**	**70**
	**Hypermethylated**	**0**		**0**		**5**	**7.7**	**2**	**10**	**29**	**32.2**	**6**	**30**
***ESR1***	**Hypomethylated**	**0**	**0**	**0**	**0**	**0**		**0**		**0**		**0**	**0**
	**Normal like**	**6**	**100**	**26**	**100**	**63**	**97**	**19**	**95**	**63**	**98.4**	**19**	**100**
	**Hypermethylated**	**0**		**0**		**2**	**3**	**1**	**5**	**1**	**1.6**	**0**	
***FOXC1***	**Hypomethylated**	**0**	**0**	**0**		**0**		**0**		**0**		**0**	
	**Normal like**	**6**	**100**	**6**	**23**	**34**	**52.3**	**7**	**35**	**40**	**46.5**	**10**	**47.6**
	**Hypermethylated**	**0**		**20**	**77**	**31**	**47.7**	**13**	**65**	**46**	**53.5**	**11**	**52.4**
***GSTP1***	**Hypomethylated**	**0**	**0**	**1**	**3.8**	**0**		**0**		**0**		**0**	
	**Normal like**	**6**	**100**	**10**	**38.5**	**27**	**41.5**	**5**	**25**	**29**	**33**	**5**	**25**
	**Hypermethylated**	**0**		**15**	**57.7**	**38**	**58.5**	**15**	**75**	**59**	**67**	**15**	**75**
***IGF2***	**Hypomethylated**	**0**	**0**	**11**	**42.3**	**25**	**38.4**	**6**	**30**	**34**	**39.1**	**9**	**47.4**
	**Normal like**	**6**	**100**	**14**	**53.8**	**32**	**49.2**	**9**	**45**	**32**	**36.8**	**5**	**26.3**
	**Hypermethylated**	**0**	**0**	**1**	**3.9**	**8**	**12.4**	**5**	**25**	**21**	**24.1**	**5**	**26.3**
***MGMT***	**Hypomethylated**	**0**	**0**	**0**		**13**	**20.3**	**13**	**65**	**16**	**24.2**	**8**	**44.4**
	**Normal like**	**6**	**100**	**25**	**96.1**	**50**	**78.1**	**5**	**25**	**35**	**53.1**	**5**	**27.7**
	**Hypermethylated**	**0**		**1**	**3.9**	**1**	**11.6**	**2**	**10**	**15**	**22.7**	**5**	**27.7**
***MLH1***	**Hypomethylated**	**0**	**0**	**4**	**15.4**	**12**	**18.5**	**6**	**32**	**4**	**4.5**	**3**	**17.6**
	**Normal like**	**6**	**100**	**21**	**80.8**	**47**	**72.3**	**10**	**53**	**68**	**77.3**	**13**	**76.5**
	**Hypermethylated**	**0**		**1**	**3.8**	**6**	**9.2**	**3**	**16**	**16**	**18.2**	**1**	**5.9**
***PPP2R2B***	**Hypomethylated**	**0**	**0**	**0**		**0**		**0**		**0**		**0**	
	**Normal like**	**6**	**100**	**7**	**26.9**	**15**	**24.6**	**6**	**35**	**40**	**30.8**	**3**	**16.7**
	**Hypermethylated**	**0**		**19**	**73.1**	**46**	**75.4**	**11**	**65**	**45**	**69.2**	**15**	**83.3**
***PTEN***	**Hypomethylated**	**0**	**0**	**0**		**0**		**0**		**0**		**0**	
	**Normal like**	**6**	**100**	**21**	**80.8**	**56**	**86.2**	**13**	**65**	**23**	**27.7**	**9**	**45**
	**Hypermethylated**	**0**		**5**	**19.2**	**9**	**13.8**	**7**	**35**	**60**	**72.3**	**11**	**55**
***RASSF1A***	**Hypomethylated**	**0**	**0**	**2**	**7.7**	**2**	**3.1**	**1**		**0**		**0**	
	**Normal like**	**6**	**100**	**2**	**7.7**	**10**	**15.4**	**2**	**11**	**3**	**4.8**	**1**	**5.6**
	**Hypermethylated**	**0**		**22**	**84.6**	**53**	**81.5**	**17**	**90**	**60**	**95.2**	**17**	**94.4**

Samples were considered as hypermethylated if the% DNA methylation was higher than the sum of two times the standard deviation and mean of the normal samples and hypomethylated if% DNA methylation was lower than two times the standard deviation - mean of the normal samples.

**Figure 1:**
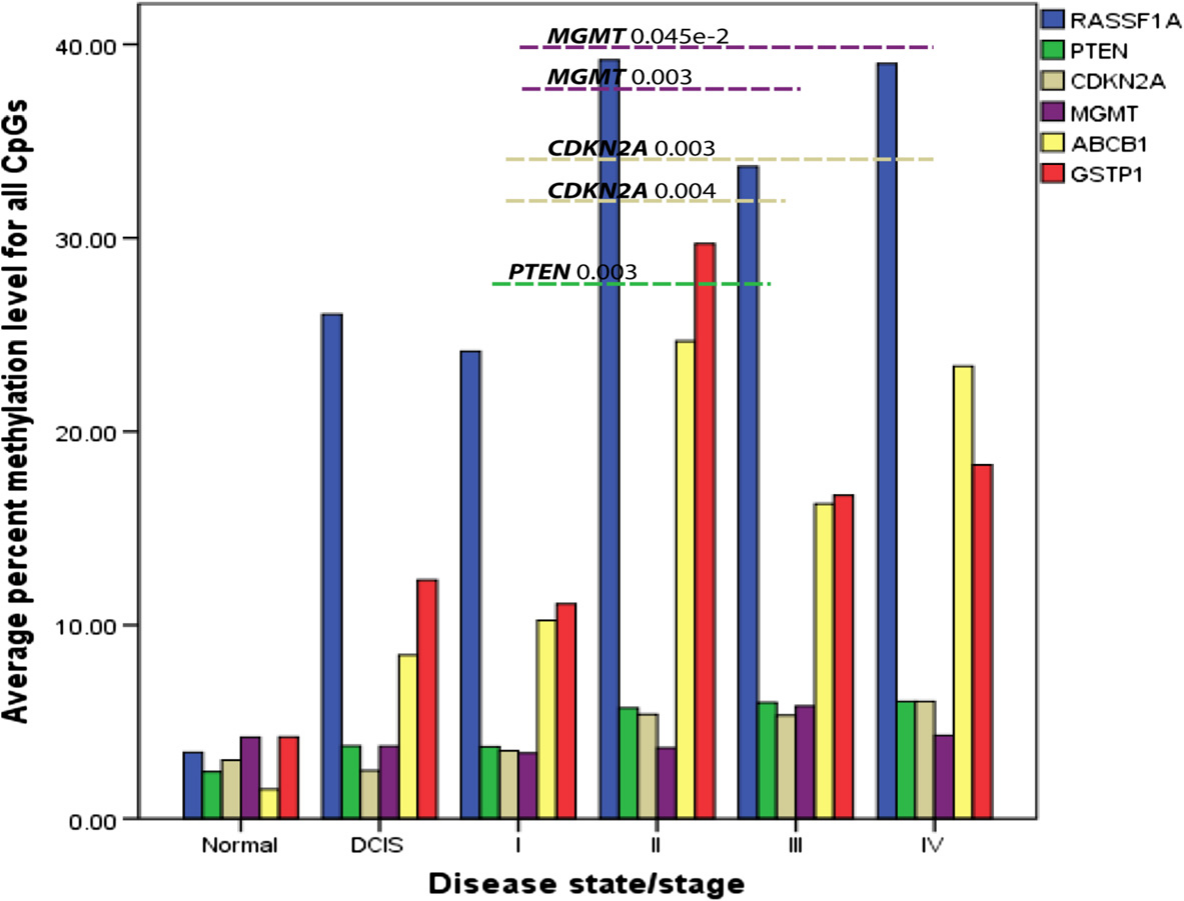
Average percentage of DNA methylation levels for all CpGs between normal, DCIS and invasive tumor samples (stage I, II, III and IV).

**Figure 2:**
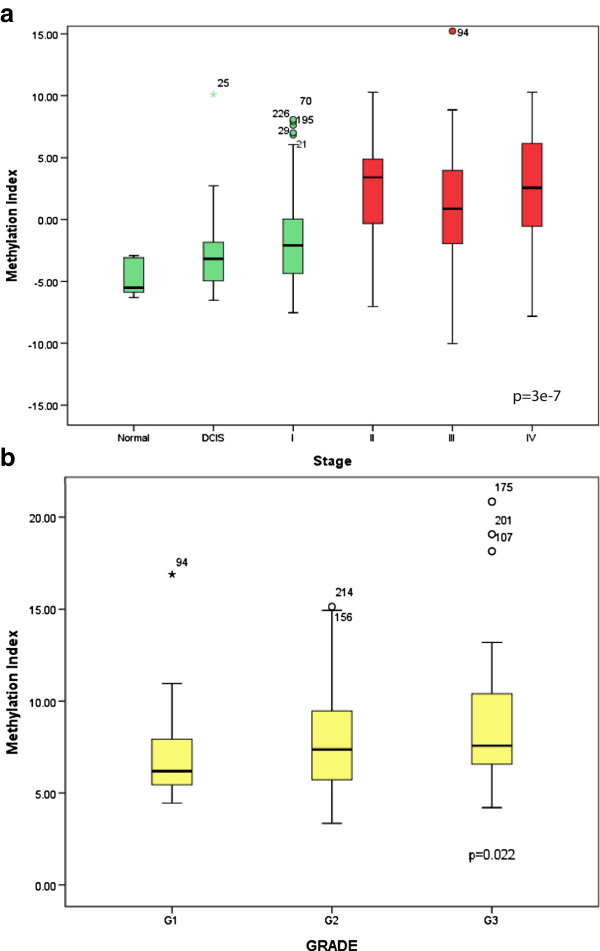
**Boxplots illustrating significant association between methylation status and tumor stage and grade. (a)** Significantly lower methylation levels in DCIS and stage I samples compared to stage II, III and IV samples was observed. **(b)** Also grade 1 tumors had significantly lower methylation index compared with grade 2 and grade 3 tumors.

Significant differences in methylation levels between the DCIS and invasive stage II tumors were observed for six genes *RASSF1A, CDKN2A, MGMT, ABCB1, GSTP1* and *FOXC1* (p = 0.008, p = 0.005, p = 0.003, p = 0.006, p = 0.010, p = 0.010 respectively). *RASSF1A*, *ABCB1* and *GSTP1* showed significantly higher quantitative methylation levels in late stage compared to the early stage breast carcinoma. The most significant differences in methylation levels for these three genes were between stage I and III (p = 0.001, p = 0.022, p = 0.019 respectively) and between stage I and IV (p = 3.3e-6, p = 0.030 and p = 0.014 respectively). *PTEN* and *CDKN2A* methylation levels were low and increased in late stage III and IV (p = 0.003, p = 0.004 between stage I and III, p = 0.018 and p = 0.003 between stage I and IV), while *MGMT* methylation levels were low and appear to decrease with tumor stage (p = 4.5e-4 between stage I and IV) (Figure 1). After correction for multiple testing (Bonferroni correction), differences in methylation levels for *RASSF1A* between stage I and III and stage I and IV remained significant. Absolute differences in mean methylation levels for *RASSF1A* were higher than 10%. For *PTEN, CDKN2A, MGMT* methylation levels between stage I and III and stage I and IV remained significant after correction. Differences in methylation levels between the DCIS and invasive stage II tumors for *MGMT* also reached statistical significance after correction. Absolute differences in mean methylation levels between different stages for *PTEN, CDKN2A, MGMT* were less than 3% even though they remained significant after Bonferroni correction. We next combined all the methylation data into a single variable, the methylation index, to investigate further, how methylation levels change during progression of breast cancer. At the same time we wanted to investigate the presence of general pattern which might be more robust than single genes. As shown in Figure 2, we observed significantly lower methylation levels in DCIS and stage I samples compared to stage II, III and IV samples (p = 3e-7). Significant differences in methylation levels were observed between the normal breast tissue and stage II, III and IV tumors (p = 0.001, p = 0.006, p = 0.009 respectively). Normal breast tissue showed lower levels of methylation compared with tumors mentioned above. There was no significant difference observed between the normal samples and the DCIS and stage I tumors. Also grade 2 and 3 tumors had significantly higher Z-scores than grade 1 tumors (p = 0.022). Methylation index analysis showed that Luminal A and Luminal B tumors had significantly higher Z-scores than Basal-like tumors (p = 0.007).

### Correlation with *TP53* mutations and hormone receptor status

**Figure 3:**
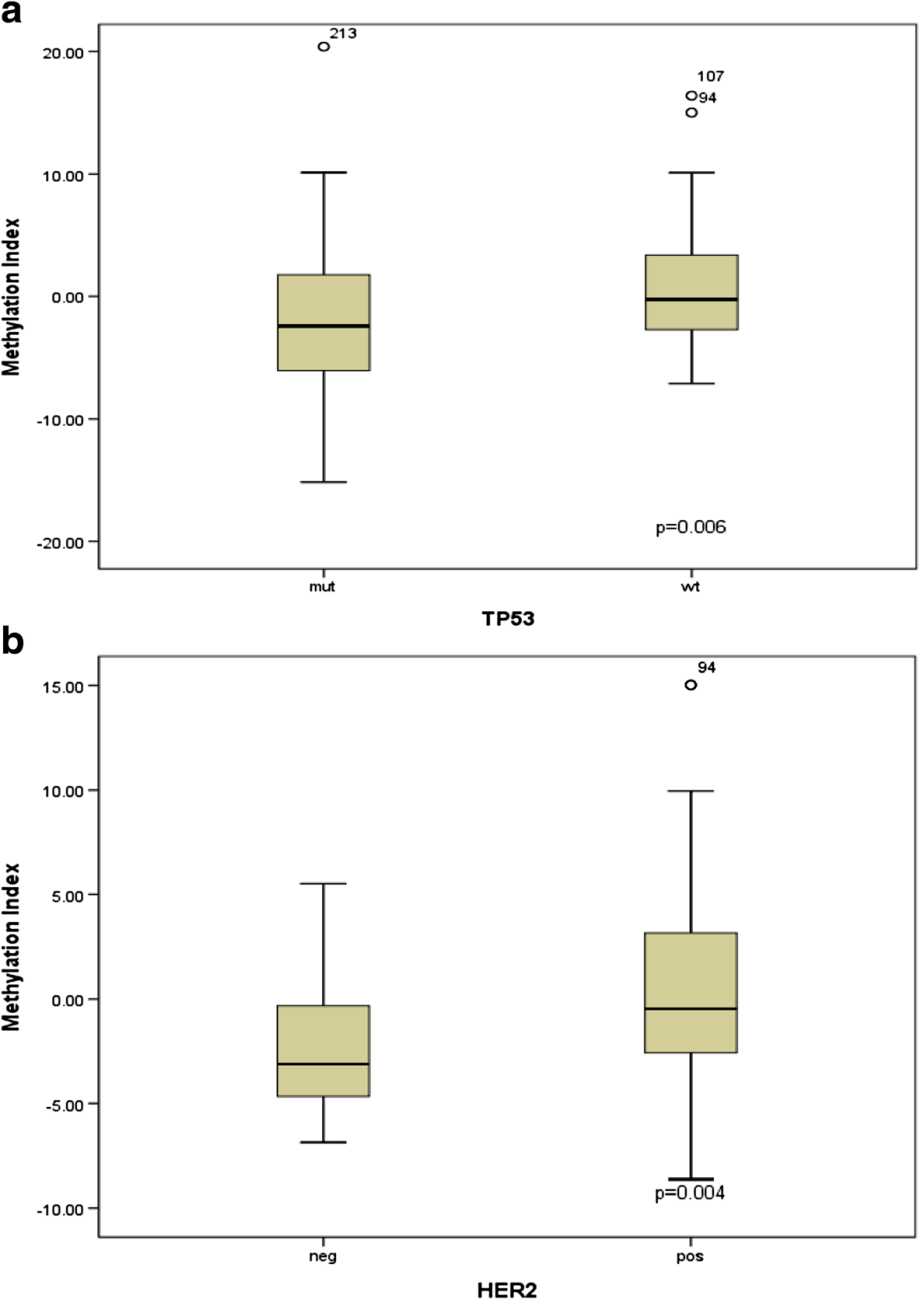
**Boxplots illustrating significant association between methylation status and HER2 and *TP53 *status. (a)***TP53* mutated tumors had significantly lower overall methylation levels compared to tumors with wild type *TP53*. **(b)** HER2 positive tumors had significantly higher methylation index compared to the HER2 negative tumors.

We compared the DNA methylation profiles with the *TP53* mutations status and found that tumors with *TP53* mutations had significantly lower DNA methylation levels then tumors with *TP53* wild type in *RASSF1A, PTEN, PPP2R2B, FOXC1, ABCB1* and *BRCA1* (p = 0.028, p = 0.031, p = 0.002, p = 0.017, p = 0.010, p = 0.001 respectively). After Bonferroni correction DNA methylation levels in *PPP2R2B* and *BRCA1* were still significantly lower in tumors with *TP53* mutations. Significant associations with (ER) estrogen receptor status were observed for *RASSF1A*, *PPP2R2B*, *GSTP1* and *FOXC1* methylation levels (p = 0.004, p = 0.012, p = 0.012, p = 0.032, respectively). HER2 receptor status was associated with *RASSF1A*, *PTEN, MGMT, CDKN2A* and *ESR1* (p = 0.023, p = 3.6e-7, p = 1.1e-8, p = 5.8e-9, p = 0.017) and after Bonferroni correction *PTEN, MGMT* and *CDKN2A* remained significant. No significant association with (PR) progesterone receptor status was observed (Table 4). Mann–Whitney test revealed that ER and HER2 negative tumors had lower methylation levels compared with ER and HER2 positive tumors for all studied genes. Interestingly HER2 negative tumors had higher methylation levels of *ESR1* compared with HER2 positive tumors (p = 0.017). Z-score analysis showed that *TP53* mutated tumors had significantly lower overall methylation levels compared to tumors with wild type *TP53*. Also HER2 positive tumors had significantly higher z-scores of methylation compared to the HER2 negative tumors (Figure 3). There was no significant association with PR and ER status.

**Table 4:** **Associations between methylation status and *TP53 *mutation and hormone receptor status**

**GENE**	**TP53 wt/mut p-value**			**ER pos/neg p-value**	**HER2 pos/neg p-value**
	**WT% meth**	**Mut% meth**	**p-value**		
*BRCA1*	87.34	79.99	0.001		
*PTEN*	5.34	4.67	0.031		3.6e-6
*PPP2R2B*	11.33	9.47	0.002	0.012	
*FOXC1*	17.12	13.56	0.017	0.032	
*ABCB1*	14.52	10.28	0.010		
*RASSF1A*	32.48	26.58	0.028	0.004	0.023
*GSTP1*				0.012	
*CDKN2A*					5.8e-9
*MGMT*					1.1e-7
*ESR1*					0.017

Indicated are % methylation within the studied subgroups together with p-values for differences in DNA methylation levels between genes in connection with ER, HER2 and TP53 mutation status. Mann–Whitney test.

### Survival analysis

To investigate which parameters contribute to differences in survival we applied: 1) univariate analysis using Kaplan-Meier modeling and the log-rank test for each gene and each clinical parameter and 2) multivariate analysis using Cox hazard proportional model to all variables. To select the best-fitted model from all model candidates, we evaluated the Akaike Information Criterion [17].

**Figure 4:**
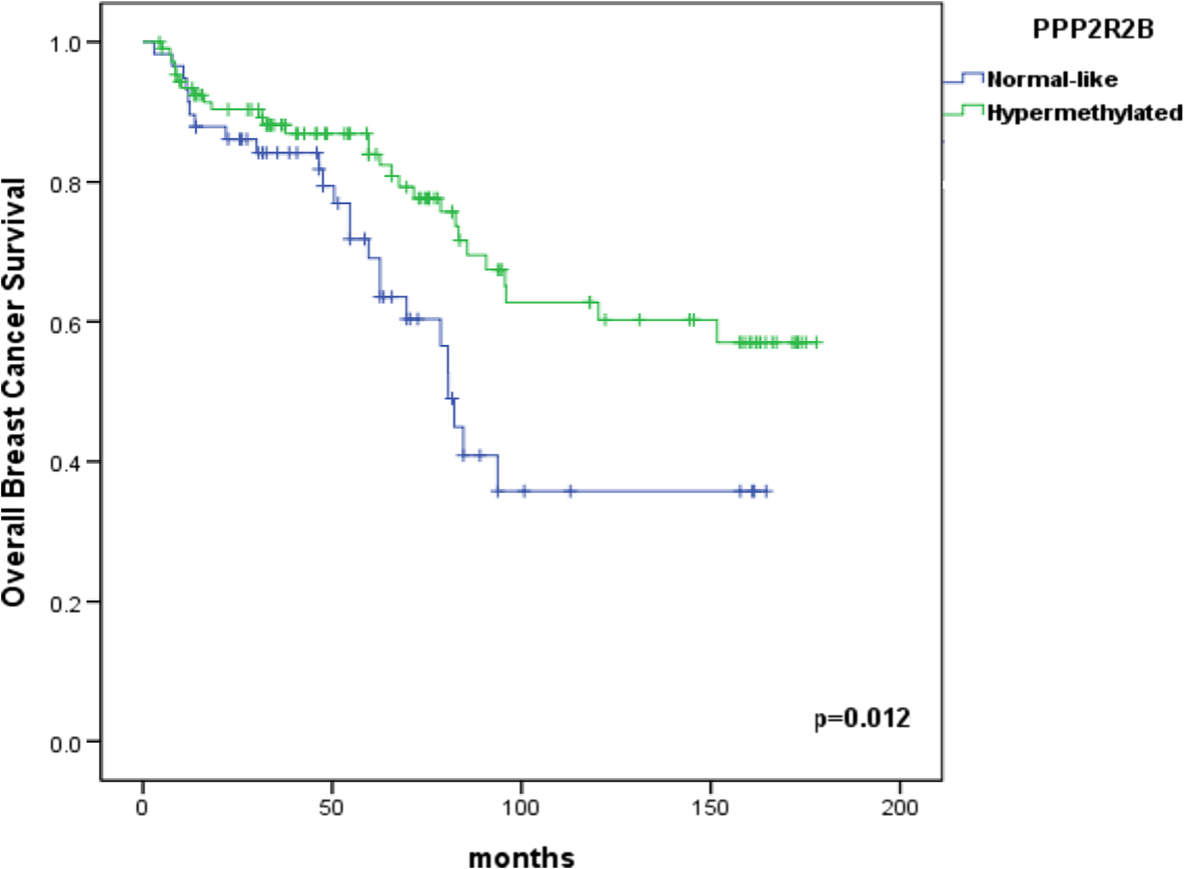
**Kaplan-Meier plots of overall survival for patients with normal-like or hyper methylated *PPP2R2B *promoter.** The p value was calculated using a long-rank test.

Univariate survival analysis identified methylation status of *PPP2R2B* as significant predictor of overall survival. As expected, grade, estrogen receptor status, *TP53* status and stage also appeared as significant predictors of survival. The Kaplan-Meier plot (Figure 4) showed a significant difference in survival between hypermethylated and normal-like samples for *PPP2R2B* (p = 0.012) indicating that patients with hypermethylated genes had better survival. Breast cancer specific survival was significantly improved in patients with hyper-methylated promoters for *PPP2R2B* (p = 0.012).

Further, multivariate survival analysis was performed in order to investigate if any of the methylation markers were independent prognostic markers, using both, categorical and continuous methylation data. We constructed possible model candidates using all relevant parameters and methylation data. Using calculated AIC, the best model that fits to the survival data was assessed. For categorical methylation data AIC identified a model explaining survival, which included the methylation status of *IGF2, GSTP1,* estrogen receptor status, *TP53*, N status and stage. The estimated coefficients, the hazard ratio, the p-values, and the 95% confidence intervals of the hazard ratio were summarized in Table 5. For continuous methylation data the best model explaining survival included TP53, T status, N status, estrogen receptor status and methylation status of *IGF2* and *GSTP1.* The estimated coefficients, the hazard ratio, the p-values, and the 95% confidence intervals of the hazard ratio were summarized in Table 6. For both, categorical and continuous methylation data *IGF2* and *GSTP1* (p = 0.009 and p = 0.014) were significant together with ER status (p = 0.008), *TP53* status (p = 6.6e-5) and N status (p = 0.047). For these models, we also described statistical significance for the likelihood ratio, Wald and log-rank tests in the Tables 5 and 6.

**Table 5:** Multivariate survival analysis - categorical methylation data

**Covariate**	**Baseline**	**Coefficient(b**_**i**_**)**	**HR(exp(b**_**i**_**)**	**p-value**	**95,0%CI for Exp(B)**
*TP53*	TP53 wild type	−2.332	0.097	6.6e-5	(0.030-0.305)
ER	ER positive	1.481	4.401	0.008	(1.460-13.269)
Stage II	Stage I	2.260	9.584	0.013	(1.593-57.667)
Stage III	Stage I	−0.011	0.988	0.992	(0.088-11.019)
N1	N0	−6.362	0.529	0.338	(0.143-1.947)
N2	N0	1.673	5.332	0.047	(1.019-27.885)
*IGF2*	Normal like *IGF2*	1.597	4.940	0.009	(1.472-16.581)
*GSTP1*	Normal like *GSTP1*	−1.621	0.197	0.014	(0.053-0.729)
*PPP2R2B*	Normal like *PPP2R2B*	−0.972	0.378	0.085	(0.125-1.143)
*CDKN2A*	Normal like *CDKN2A*	−0.839	0.432	0.133	(0.144-1.294)

The variables that the minimum AIC selected. ( p-values: 3.4e-7 for likelihood ratio test, 3.4e-4 for Wald test, 3.2e-6 for log-rank test).

Positive hazard ratios indicate an increased risk of dying from breast cancer and are calculated for the different covariates.

**Table 6:** Multivariate survival analysis - continuous methylation data

**Covariate**	**Baseline**	**Coefficient (b**_**i**_**)**	**HR(exp(b**_**i**_**)**	**p-value**	**95,0% CI for Exp(B)**
*GSTP1*	Normal like *GSTP1*	−0.066	0.935	4e-4	(0.902-0.970)
*IGF2*	Normal like *IGF2*	0.079	1.082	0.002	(1.031-1.136)
*MLH1*	Normal like *MLH1*	−0.552	0.575	0.135	(0.278-1.187)
ER	ER positive	1.659	5.256	0.002	(1.804-15.317)
*TP53*	TP53 wild type	−2.169	0.114	0.002	(0.028-0.450)
N 1	N0	1.684	5.391	0.022	(1.265-22.975)
N 2	N0	1.706	5.511	0.032	(1.151-26.371)
T 2	T1	1.937	6.942	0.035	(1.143-42.157)
T3	T1	0.050	1.051	0.960	(0.143-7.716)
T4	T1	1.125	3.081	0.258	(0.437-21.692)

The variables that the minimum AIC selected. ( p-values: 1.2e-7 for likelihood ratio test, 5.8e-4 for Wald test, 6.7e-6 for log-rank test).

Positive hazard ratios indicate an increased risk of dying from breast cancer and are calculated for the different covariates.

## Discussion

The aim of this study was to quantitatively determine the methylation levels in the promoter region of 12 genes in breast cancer patients of all stages and to investigate stage specific changes in tumors. In addition, we wanted to evaluate the association between clinico-pathological factors (ER, HER2 status, grade) including survival and methylation levels of these genes.

In the present study, five genes, *ABCB1, FOXC1*, *GSTP1, PPP2R2B* and *RASSF1A* were hypermethylated already in early stage breast cancer (stage I and II). For all five genes hypermethylation was also detected in DCIS suggesting that inactivation of these genes is a frequent event in the process of mammary tumorigenesis. We found that *RASSF1A* was hypermethylated in approximately 85% of all invasive tumors and DCIS, and our results are in agreement with such a high incidence of *RASSF1A* methylation [18-20]. *RASSF1A* is a putative tumor-suppressor gene. It belongs to an increasing list of tumor suppressor genes that are frequently inactivated by promoter methylation rather than by somatic mutations [21]. Since we detected a constant hypermethylation of *RASSF1A* in all of the different stages of the breast carcinomas we can suggest that hypermethylation of *RASSF1A* is an early event during breast cancer pathogenesis and also the main mechanism of inactivation. In our study, *GSTP1* was found to be hypermethylated in different stages of breast carcinomas, for early stages (I and II) our results are in agreement with previous reports [22,23]. The frequency of *GSTP1* promoter hypermethylation in stage III and IV (around 70%) was found to be higher than reported previously [23,24]. It is known that *GSTP1* plays a role in detoxification of potential carcinogens and that loss of the expression of *GSTP1* will lead to DNA damage of breast cells and they will be more easily exposed to carcinogens [25]. Loss of *GSTP1* expression and its potential role in breast carcinogenesis was observed in high proportion of breast tumors [23]. It appears that promoter hypermethylation is associated with loss of *GSTP1* expression [26]. Important question is when the promoter hypermethylation of the *GSTP1* gene starts to play a role in tumor progression? We found that already in DCIS there is a high proportion of hypermethylated *GSTP1* (58%), which indicates that *GSTP1* promoter hypermethylation is an early event in breast carcinogenesis. From our analysis we observed that hypermethylation of *ABCB1, PPP2R2B* and *FOXC1* is also an early event in breast carcinogenesis since our results indicate high level of hypermethylation of these genes already in DCIS which was reported before [4]. We found frequencies of methylation for *CDKN2A MGMT* and *MLH1* similar to previously published reports [27,28]. According to our results, we could suggest that hypermethylation of *CDKN2A* is possible event leading to its inactivation in late stage breast cancers. *PTEN* is a tumor suppressor gene. Its product PTEN protein works as a negative regulator of the Akt pathway, leading to suppression of apoptosis and increasing cell survival [29]. We suggest here that epigenetic silencing of *PTEN* might be an early event in initiation of cancer and also the mechanism of its inactivation. Next, combining all the methylation data into a single variable, the methylation index, we investigate how methylation levels change during progression of breast cancer. Our analysis showed that the methylation pattern of all genes included in this study is changing during breast cancer progression. DCIS and stage I tumors had similar methylation levels with no significant difference. Stage II tumors showed the most significant difference in methylation levels when compared with DCIS and stage I and then in stage III and IV methylation levels were lower but still significantly higher than in DCIS and stage I. It remains unclear how this process of dramatic change in stage II is achieved and driven further during breast cancer progression. To our knowledge only one group investigate stage dependent DNA methylation in breast cancer using DCIS and all four stages of invasive tumors [30]. They identified 33 cancer specific genes that were either highly methylated in early stage breast cancer or showed stage dependent methylation pattern, lower methylation frequency in early stage breast cancers and a higher methylation frequency in late stage breast cancers. None of our selected genes were methylated in this study. Additional studies of the relationship between DNA methylation in tumors and tumor stages are necessary.

In the present study we showed the associations between DNA methylation levels of candidate genes and the *TP53* mutation status, estrogen receptor status, and HER2 status. The *TP53* tumor suppressor gene has a central role in cell cycle regulation, DNA repair and apoptosis, and a large number of reports have discussed the important role of *TP53* alterations in breast cancer. Also, a number of studies have shown that breast tumors with *TP53* mutations are strongly associated with poor prognosis and lacking methylation in a number of regulatory genes [31,32]. Additionally, studies on different expression subtypes in breast cancer showed that different subtypes have a different underlying biology reflected in methylation and is strongly influenced by *TP53* mutation status. It was shown that basal-like tumors are *TP53* mutated and unmethylated [6,33]. In present study we have identified 26,6% of breast tumors with *TP53* mutations and significantly lower levels of DNA methylation in *RASSF1A, PTEN, PPP2R2B, FOXC1, ABCB1* and *BRCA1* compared to tumors with wild type *TP53*. Since in our study we had low number of basal-like tumors (37 out of 238) we cannot confirm association between *TP53* mutated and unmethylated tumors and basal-like but most of the basal-like samples were *TP53* mutated and had lowest methylation levels compared to other subtypes (data not shown). The status of ER and HER2 have been recognized as important prognostic factors in patients with breast cancer, in addition to a predictive marker for the response to treatment with endocrine and trastuzumab therapy. Identification of genes with subtype-specific methylation revealed that *RASSF1A* and *GSTP1* were highly methylated in Lum B tumors [33]. These two genes were reported previously to be significantly more methylated in ER- positive than ER-negative tumors [34]. In the present study we showed the same trend in ER-positive tumors compared to ER-negative for four genes and *RASSF1A* and *GSTP1* were among them. The same group reported that HER2 positive tumors had higher methylation level for these two genes compared to HER2-negative tumors, which is again in accordance with our study. Furthermore, in a study on methylation in breast cancer and breast cancer molecular subtypes it was shown that *RASSF1A* is hypermethylated in HER2 positive breast tumors (ERBB2 and luminal B) [35]. In our study *RASSF1A* was hypermethylated in ERBB2 and luminal B tumors (data not shown). Taken all together, these results, suggest that methylation plays a significant role in the different breast tumor phenotypes.

We report here for the first time the *PPP2R2B* methylation status as significant predictor for breast cancer survival as well as for overall survival. *PPP2R2B* is a candidate tumor suppressor gene and it was shown that changes in DNA methylation of this gene contribute to its expression [36]. Further, multivariate analysis showed that *IGF2* and *GSTP1* were independent prognostic markers. Recently, it has been shown that the absence of GSTP1 protein expression correlate with promoter hypermethylation and with improved survival in invasive breast cancer samples [37]. Hypermethylation of *GSTP1* is a well established biomarker for hormone dependent cancers. Our previous analyses suggest that methylation of *GSTP1* in locally advanced breast cancer patients treated with doxorubicin was associated to survival [3]. However, GSTP1 had no effect on treatment response.

## Conclusions

Here we report aberrant methylation levels of *ABCB1, FOXC1, GSTP1, PPP2R2B* and *RASSF1A* in DCIS and stage I-IV providing evidence that suggests that changes in methylation level is an early event and may also be important in progression to later stages of breast cancer. We also report that methylation levels of important breast cancer genes are associated to hormone receptor status and TP53 mutation status suggesting mechanisms of deactivation of tumor suppressor genes in breast cancer. Further studies are necessary to identify which methylation gene profiles are of predictive and which of prognostic value.

## Competing interests

The authors declare that they have no competing interests.

## Authors’ contribution

JK performed data analyses and wrote the manuscript. ED perfomed laboratory experiments.TF, HE and HS were involved in the statistical analyses. PEL and FW were responsible for the patient cohorts and IB for the control samples. ALBD, JT and VNK initiated and designed the study and participated in writing the manuscript. All authors have read and approved the final manuscript.

## Pre-publication history

The pre-publication history for this paper can be accessed here:

http://www.biomedcentral.com/1471-2407/13/456/prepub

## Supplementary Material

Additional file 1 **PCR and pyrosequencing primers.** Sequences of primers used for amplification and pyrosequencing reactions, Genbak accession numbers and nucleotides (Nt) corresponding to the amplified fragments as well as the annealing temperatures for the respective PCR amplifications. CpGs are numbered in the order of appearance from the 5' end of an amplification product. Y = pyrimidine. Click here for file

## References

[B1] Jovanovic J Rønneberg JA Tost J Kristensen V The epigenetics of breast cancer Mol Oncol 2010 4 242 254 10.1016/j.molonc.2010.04.002 20627830 PMC5527941

[B2] Jones PA Baylin SB The epigenomics of cancer Cell 2007 128 683 692 10.1016/j.cell.2007.01.029 17320506 PMC3894624

[B3] Dejeux E Rønneberg JA Solvang H Bukholm I Geisler S Aas T Gut IG Børresen-Dale AL Lønning PE Kristensen VN Tost J DNA methylation profiling in doxorubicin treated primary locally advanced breast tumours identifies novel genes associated with survival and treatment response Mol Cancer 2010 9 68 10.1186/1476-4598-9-68 20338046 PMC2861056

[B4] Muggerud AA Rønneberg JA Wärnberg F Botling J Busato F Jovanovic J Solvang H Bukholm I Børresen-Dale AL Kristensen VN Sørlie T Tost J Frequent aberrant DNA methylation of ABCB1, FOXC1, PPP2R2B and PTEN in ductal carcinoma in situ and early invasive breast cancer Breast Cancer Res 2010 12 R3 10.1186/bcr2466 20056007 PMC2880421

[B5] Geisler S Børresen-Dale AL Johnsen H Aas T Geisler J Akslen LA Anker G Lønning PE TP53 gene mutations predict the response to neoadjuvant treatment with 5-fluorouracil and mitomycin in locally advanced breast cancer Clin Cancer Res 2003 9 5582 8 14654539

[B6] Rønneberg JA Fleischer T Solvang HK Nordgard SH Edvardsen H Potapenko I Nebdal D Daviaud C Gut I Bukholm I Naume B Børresen-Dale AL Tost J Kristensen V Methylation profiling with a panel of cancer related genes: association with estrogen receptor, TP53 mutation status and expression subtypes in sporadic breast cancer Mol Ocol 2011 5 61 76 10.1016/j.molonc.2010.11.004 PMC5528272 21212030

[B7] Tost J Schatz P Schuster M Berlin K Gut IG Analysis and accurate quantification of CpG methylation by MALDI mass spectrometry Nucleic Acids Res 2003 31 e50 10.1093/nar/gng050 12711695 PMC154238

[B8] David GL Yegnasubramanian S Kumar A Marchi VL De Marzo AM Lin X Nelson WG MDR1 Promoter hypermethylation in MCF-7 human breast cancer cells: changes in chromatin structure induced by treatment with 5-Aza-cytidine Cancer Biol Therap 2004 3 540 548 15034303 10.4161/cbt.3.6.845

[B9] Esteller M Corn PG Baylin SB Herman JG A gene hypermethylation profile of human cancer Cancer Res 2001 61 3225 3229 11309270

[B10] Lapidus RG Nass SJ Butash KA Parl FF Weitzman SA Graff JG Herman JG Davidson NE Mapping of ER gene CpG island methylation-specific polymerase chain reaction Cancer Res 1998 58 2515 2519 9635570

[B11] Esteller M Corn PG Urena JM Gabrielson E Baylin SB Herman JG Inactivation of glutathione S-transferase P1 gene by promoter hypermethylation in human neoplasia Cancer Res 1998 58 4515 4518 9788592

[B12] Issa JP Vertino PM Boehm CD Newsham IF Baylin SB Switch from monoallelic to biallelic human IGF2 promoter methylation during aging and carcinogenesis Proc Natl Acad Sci U S A 1996 93 11757 11762 10.1073/pnas.93.21.11757 8876210 PMC38131

[B13] Keen JC Garrett-Mayer E Pettit C Mack KM Manning J Herman JG Davidson NE Epigenetic regulation of protein phosphatase 2A (PP2A), lymphotactin (XCL1) and estrogen receptor alpha (ER) expression in human breast cancer cells Cancer Biol Therap 2004 3 1304 1312 10.4161/cbt.3.12.1458 15662126

[B14] Garcia JM Silva J Pena C Garcia V Rodriguez R Cruz MA Cantos B Provencio M Espana P Bonilla F Promoter methylation of the PTEN gene is a common molecular change in breast cancer Genes Chromosomes Cancer 2004 41 117 124 10.1002/gcc.20062 15287024

[B15] Yan PS Shi H Rahmatpanah F Hsiau TH Hsiau AH Leu YW Liu JC Huang TH Differential distribution of DNA methylation within the RASSF1A CpG island in breast cancer Cancer Res 2003 63 6178 6186 14559801

[B16] Sorlie T Perou CM Tibshirani R Aas T Geisler S Johnsen H Hastie T Eisen MB van de Rijn M Jeffrey SS Thorsen T Quist H Matese JC Brown PO Botstein D Lonning PE Borresen-Dale AL Gene expression patterns of breast carcinomas distinguish tumor subclasses with clinical implications Proc Natl Acad Sci U S A 2001 98 10869 10874 10.1073/pnas.191367098 11553815 PMC58566

[B17] Akaike H A new look at the statistical identification model IEEE Trans Automatic Control 1974 19 716 723 10.1109/TAC.1974.1100705

[B18] Fackler MJ McVeigh M Evron E Garrett E Mehrotra J Polyak K Sukumar S Argani P DNA methylation of RASSF1A, HIN-1, RAR-β, cyclin D2 and twist in in situ and invasive lobular breast carcinoma Int J Cancer 2003 107 970 975 10.1002/ijc.11508 14601057

[B19] Yeo W Wong WL Wong N Law BK Tse GM Zhong S High frequency of promoter hypermethylation of RASSF1A in tumorous and non-tumourous tissue of breast cancer Pathology 2005 37 125 130 10.1080/00313020500058623 16028839

[B20] Kioulafa M Kaklamanis L Mavroudis D Georgoulias V Lianidou ES Prognostic significance of RASSF1A promoter methylation in operable breast cancer Clin Biochem 2009 42 970 975 10.1016/j.clinbiochem.2009.04.003 19374895

[B21] Jones PA Baylin SB The fundamental role of epigenetic events in cancer Nat Rev Genet 2002 3 415 428 12042769 10.1038/nrg816

[B22] Lee JS GSTP1 promoter hypermethylation is an early event in breast carcinogenesis Virchows Arch 2007 450 637 642 10.1007/s00428-007-0421-8 17479284

[B23] Arai T Miyoshi Y Kim SJ Taguchi T Tamaki Y Noguchi S Association of GSTP1 CpG islands hypermethylation with poor prognosis in human breast cancers Breast Cancer Res Treat 2006 100 169 176 10.1007/s10549-006-9241-9 16791478

[B24] Hoque MO Feng Q Toure P Dem A Critchlow CW Hawes SE Wood T Jeronimo C Rosenbaum E Stern J Yu M Trink B Kiviat NB Sidransky D Detection of aberrant methylation of four genes in plasma DNA for the detection of breast cancer J Clin Oncol 2006 24 4262 4269 10.1200/JCO.2005.01.3516 16908936

[B25] Lee WH Morton RA Epstein JI Brooks JD Campbell PA Bova GS Hsieh WS Isaacs WB Nelson WG Cytidine methylation of regulatory sequences near the pi-class glutathione S-transferase gene accompanies human prostatic carcinogenesis Proc Natl Acad Sci U S A 2004 91 11733 11737 7972132 10.1073/pnas.91.24.11733 PMC45306

[B26] Zhong S Tang MW Yeo W Liu C Lo YM Johnson PJ Silencing of GSTP1 gene by CpG island DNA hypermethylation in HBV-associated hepatocellular carcinomas Clin Cancer Res 2002 8 1087 1092 11948118

[B27] Nielsen NH Roos G Emdin SO Landberg G Methylation of the p16 (Ink4a) tumor suppressor gene 5'-CpG island in breast cancer Cancer Lett 2001 163 59 59 10.1016/S0304-3835(00)00674-1 11163109

[B28] Viswanathan M Solomon SP Tsuchida N Selvam GS Shanmugam G Methylation of E-cadherin and hMLH1 genes in Indian sporadic breast carcinomas Indian J Exp Biol 2006 44 115 119 16480176

[B29] Khan S Kumagai T Vora J Bose N Sehgal I Koeffler PH Bose S *PTEN* promoter is methylated in a proportion of invasive breast cancers Int J Cancer 2004 112 407 410 10.1002/ijc.20447 15382065

[B30] Jeschke J Van Neste L Glöckner SC Dhir M Calmon MF Deregowski V Van Criekinge W Vlassenbroeck I Koch A Chan TA Cope L Hooker CM Schuebel KE Gabrielson E Winterpacht A Baylin SB Herman JG Ahuja N Biomarkers for detection and prognosis of breast cancer identified by a functional hypermethylome screen Epigenetics 2012 7 701 709 10.4161/epi.20445 22647880

[B31] Feki A Irminger-Finger I Mutational spectrum of p53 mutations in primary breast and ovarian tumors Crit Rev Oncol Hematol 2004 52 103 116 10.1016/j.critrevonc.2004.07.002 15501075

[B32] Feng W Shen L Wen S Rosen DG Jelinek J Hu X Huan S Huang M Liu J Sahin AA Hunt KK Bast RC Jr Shen Y Issa JP Yu Y Correlation between CpG methylation profiles and hormone receptor status in breast cancers Breast Cancer Res 2007 9 R57 10.1186/bcr1762 17764565 PMC2206733

[B33] Holm K Hegardt C Staaf J Vallon-Christersson J Jönsson G Olsson H Borg A Ringnér M Molecular subtypes of breast cancer are associated with characteristic DNA methylation patterns Breast Cancer Res 2010 12 R36 10.1186/bcr2590 20565864 PMC2917031

[B34] Eiji S Masaru S Myung-Shin S Nguyen SL Anh-Thu V Giuliano AE Dave SB H Estrogen receptor and HER2/neu status affect epigenetic differences of tumor-related genes in primary breast tumors Breast Cancer Res 2008 10 R46 10.1186/bcr2098 18485221 PMC2481494

[B35] Bediaga NG Acha-Sagredo A Guerra I Viguri A Albaina C Ruiz Diaz I Rezola R Alberdi MJ Dopazo J Montaner D Renobales M Fernández AF Field JK Fraga MF Liloglou T de Pancorbo MM DNA methylation epigenotypes in breast cancer molecular subtypes Breast Cancer Res 2010 12 R77 10.1186/bcr2721 20920229 PMC3096970

[B36] Keen JC Garrett-Mayer E Pettit C Mack KM Manning J Herman JG Davidson NE Epigenetic regulation of protein phosphatase 2A (PP2A), lymphotactin (XCL1) and estrogen receptor alpha (ER) expression in human breast cancer cells Cancer Biol Ther 2004 3 1304 1312 10.4161/cbt.3.12.1458 15662126

[B37] Huang J Tan PH Thiyagarajan J Bay BH Prognostic significance of glutathione S-transferase-pi in invasive breast cancer Mod Pathol 2003 16 558 565 10.1097/01.MP.0000071842.83169.5A 12808061

