# Supplementary material for: Quantitative DNA methylation analyses reveal stage dependent DNA methylation and association to clinico-pathological factors in breast tumors
Source: BMC Cancer. 2013 Oct 5;13:456. doi: 10.1186/1471-2407-13-456 (PMC3819713; doi:10.1186/1471-2407-13-456)
Supplement: Additional file 1 — PCR and pyrosequencing primers. Sequences of primers used for amplification and pyrosequencing reactions, Genbak accession numbers and nucleotides (Nt) corresponding to the amplified fragments as well as the annealing temperatures for the respective PCR amplifications. CpGs are numbered in the order of appearance from the 5' end of an amplification product. Y = pyrimidine. [file 1471-2407-13-456-S1.pdf]

*Gene	PCR primer forward	PCR primer reverse	Tem. PCR (°C)	Pyrosequencing primer	CpG number
ABCB1_1 [GenBank:AY910577] Nt 114132-114333 -859 to -657	5' TTTTGGAAATTTAATTTGT TT	5'-Biotin-CTACTTCTTTAAACTTAA AAAAACC	53	5'- GATTGGTTGGGTAGGA	7
ABCB1_2 [GenBank:AY910577] Nt 114583-114800 -408 to -191	5' TTAGATTTAGGAGTTTTTG GAGTAG	5'-Biotin-AAAACAAAATTAAAAAT CTAACAAC	58	5'- TGGTATTGGATTATGTTGTT<br>5'- TGGGTGGGAGGAAGT	10<br>10
ABCB1_3 [GenBank:AY910577] Nt 114826-115077 -165 to +186	5' ATAGGAAAGTTTTTGTAG TGTTTAT	5'-Biotin-CAAACTAATTACCTTTTA TTATTCAATTTA	58	5'- GGTTAGAGTAGTTGGGGTA<br>5'- AGAGGTTAGTTTATTTTTA<br>5'- TAAAAATAAAACAAAA	5<br>5<br>3
BRCA1_1 [GenBank:AC135721] Nt 32624-32929 -1508 to -1202	5' GGTTAGTTTAGAGAGGGG TTTTTATAG	5'-Biotin-TCACACTAAAAAAATTTA AAAAAATTCAAA	59	5'- GGGGTTTTTATAGYGTAGTG<br>5' - GTATTTTGAATTATAGATTT<br>5'- ATTAAAAYGATTAGTTGTT	10<br>6<br>3
BRCA1_2 [GenBank:AC135721] Nt 33805-34101 -324 to -30	5' TTTTATGGTAAATTTAGGT AGAATTTT	5'-Biotin-TATCTAAAAAACCCCAC AACCTATC	58	5'- TTTGAGAGGTTGTTGTTTAG<br>5'- GGGTGGTTAATTTAGAGTTT<br>5'- GGAATTATAGATAAATTAAA	11<br>4<br>6
BRCA1_3 [GenBank:AC135721] Nt 34447-34726 +302 to +593	5' AGGAGTTTTAGATTAGTTT GATTAA	5'-Biotin-CTAACACCTCTTCTTCCA CAAAATC	58	5'- GAGTTTTAGATTAGTTTGAT<br>5'-GGAGGTTGAGGTAGGAGA	5<br>8
CDKN2A [GenBank:AF527803] Nt 19893-20154 +118to +380	5' GAGGGGTTGGTTGGTTAT TAGA	5'-biotin-TACAAACCCTCTACCCAC CTAAAT	64	5'- GAGGGGGAGAGTAGGTAG<br>5'- GGGGAGTAGTATGGAGTTTT<br>5'- GGGTYGGGTAGAGGA	6<br>8<br>9
ESR1_2 [GenBank:AY425004] Nt 2426-2736	5' GTAGTTTAAGATTTTTTTG GAG	5'-Biotin-CCAAATAATAAAACACC TACTAACC	61	5'- TATTTGGATAGTAGTAAGTT	13
+449 to +759				5'- YGTTAAYGYGTAGGTT<br>5'- TTYGTTGATGTTATTGTATT	8<br>5
ESR1_3 [GenBank:AY425004] Nt 2858-3201 +881 to +1224	5'-Biotin-GGAGAAGGGAGAGTTTAG GGAGTTG	5' ACCCCCAACTTTAAATAC CAAAAAC	66	5'- CCCCCRAACTAAACC<br>5'- AAAATACRTTTTTTTCAACT	13<br>11
FOXC1_1 [EMBL:AL034344] Nt 64529-64798 -1152 to -8568	5'-Biotin-GGTTTTTATTTTTTATTGG TTGT	5' CCTTCCAAAAAACTCTAC CCTAAAC	62	5'- GCRTCACCTTTTCTC	5
FOXC1_3 [EMBL:AL034344] Nt 65513-65727 +354 to+568	5' TAAGTAGGGTTGGTAGAA TAGTATT	5'-Biotin-ACAACCTATCCTTCTCCT CCTTATC	62	5'- TTCGGATTTTTATAATATGT	9
GSTP1_1 [GenBank:AY324387] Nt 1670-1970 +3 to +304	5' GAAAGAGGGAAAGGTTTT TT	5'-biotin-CCATACTAAAAACTCTAA ACCCCATC	58	5'- GTAGTTTTYGTTATTAGTGA<br>5'- GGATTATTTTTATAAGGT	6<br>8
GSTP1_2 [GenBank:AY324387] Nt 1670-1970 +3 to +304	5'-biotin-GAAAGAGGGAAAGGTTTT TT	5' CCATACTAAAAACTCTAA ACCCCATC	58	5'- CGAACCTTATAAAAATAATC	7
IGF2 DMR2 [GenBank:AF517226] Nt 7978-7724 +15856 to +16111	5' GGGAAAGGGGTTTAGGAT TTTTAT	5'-biotin-ATAATTTACTCCCCCTTC AACCTC	60	5'-GATTTTTATYGGAAGTA<br>5'-GGGTYGATAYGTTTTTTT<br>5'-TTTTATAGTATAGAGAGAGT	5<br>8<br>3
MLH1 [GenBank:AY217549] Nt 1504-1800 -564 to -268	5' GGGAGGTTATAAGAGTAG GGTTAA	5'-Biotin-TCTCAACTCTATAAATTA CTAAATCTCTTC	61.4	5'- GAGAGGAGGAGTTTGAGA<br>5'- TTTTTATTGGTTGGATATTT<br>5'- AAAACGAATTAATAGGAAGA	5<br>11<br>8
PPP2R2B_1 [GenBank:AF152102] Nt 1804-2020 -445 to -229	5' AGGTAGGGAGTTAGGGGT TTTG	5'-Biotin-CTCTTTCCCAACAACAAT AATAACC	62	5'- GGGGTTTTGGGTGTT<br>5'- ATTTGAATTTGTAAGTTAGT<br>5'- AATGGAGGAGGATATTGA	7<br>7<br>6
PPP2R2B_3 [GenBank:AF152102] Nt 3012-3336 +516 to +838	5' AGTTTGTTGTTAATGGAG GAGGATAT	5'-Biotin-AATCCCTAAAAACCATTT TAACTCC	62	5'-AAGGTAAYGTTAGTTT<br>5'- GGTTTTAGGGTTG<br>5'- CGGTAGTTTTAGTGTATT	11<br>10<br>10
PTEN_2 [GenBank:AF67844] Nt 22468-22791	5' ATATTGGGTATGTTTAGTA GAGTTTG	5'-Biotin-CAAACTTCCATCATAACT ACAACTTC	62	5'- TGGYGGGATTTTTTA	10
-98 to+227			62	5'- TGGYGGGATTTTTTA<br>5'-TTTGGGGATTTTG<br>5'-TTYGTATTTAGAGTTAT	10<br>3<br>12
PTEN_3 [GenBank:AF67844] Nt 22893-23149 +586 to +843	5' TTGTTATTATTTTTAGGGT TGGGAA	5'-Biotin-CTAAACCTACTTCTCCTC AACAACC	60	5'- GTTGGTATATTTAGGGATT<br>5'- CGTYGGYGGAGGTA	13<br>6
MGMT [GenBank:X61657] Nt 935-1133 -138 to +55	5' GTTTTTTTGTTTTTTTTAGGTTTT	5'-Biotin-AAACRACCCAAACACTCACC	60	5'- GGTTTYGTTTYGTTTTAGATT<br>5'- AGGATATGTTGGGATAGTT	9<br>12
RASSF1A [GenBank:DQ444319] Nt 599-789 -30 to +160	5' AGTTTTTGTATTTAGGTTTTTATTG	5'-Biotin-AACTCAATAAACTCAAACTCCCC	70.1	5'- GGATTTTGGGGGAGG<br>5'- TGTTAGYGTTTAAAGTTA	8<br>4

* Transcription start site
